# A case of neurosarcoidosis with aggresive characteristics

**DOI:** 10.1186/1546-0096-12-S1-P289

**Published:** 2014-09-17

**Authors:** Serdal Ugurlu, Ahmet Murt, Emire Seyahi, Ugur Uygunoglu, Sabahattin Saip, Huri Ozdogan

**Affiliations:** 1 Rheumatology, Cerrahpasa Medical Faculty, Istanbul, Turkey; 2 Internal Medicine, Cerrahpasa Medical Faculty, Istanbul, Turkey; 3 Neurology, Cerrahpasa Medical Faculty, Istanbul, Turkey

## Introduction

Here we are presenting a case of sarcoidosis with extensive systemic and neurologic involvement. Although different immunosuppressant modalities were used, the neurological symptoms recurred. Management of neurosarcoidosis can be challenging and needs multidisciplinary approach.

## Objectives

Presenting a case of sarcoidosis with extensive systemic and neurologic involvement.

## Methods

A male patient, who was 14 years old at that time, applied to the emergency department 4 years ago with right sided hemiplegia, right sided hemianopsia and disarticulated speech. Cranial MRI showed T2 and flair hyperintensities on optic tractus and chiasma opticum. This finding was consistent with either neoplastic diseases or aggressive inflamatory processes. Lomber punction revealed no specific finding. As the patient had pansitopenia, bone marrow aspiration / biopsy was performed. Paratrabecular granuloma was found in the bone marrow biopsy. To analyse pansitopenia and Hepatosplenomegaly, the patient underwent portal Doppler USG imaging. Splenic vein calibration seemed to be increased. A gastroscopy was performed and grade 1 esophagus varices were visualized. Abdominal CT scan showed many lymph nodes of 2 cm or larger. Hepatic biopsy showed non-necrotizing granulomatous lesions and EZN stains were negative. Abdominal lymph node biopsy was performed by a laparotomy and Schaumann bodies were seen at the microscopic analysis.

The patient was given 60 mg methylprednisolone daily and 20 mg MTX weekly. MRI after 3 weeks showed minimal regression and clinical signs of the patient ameliorated. Steroid was continued by tapering the dose and MTX was given for 6 months. As the neurological signs of the patients couldn’t be cured, patient was re-admitted after 6 months. MRI showed prominent progression in the lesions. Spinal MRI showed sequel lesions due to involvement of the spinal cord. Infliximab treatment was planned but couldn’t be continued as allergic reaction developed. Cyclophosphamid treatment was started but had to be stopped after 5 doses because of side effects (nausea, vomiting). In order to manage the progressive neurologic findings Rituximab (4 doses in total) was applied. However patient presented with recurrent neurological findings and Adalimumab treatment was given. Hepatic sarcoids were regressed in abdominal USG but gastroscopic images showed grade 2 esophagus varices. Patient had received a total of 3 doses till now and seems to have a slow regression for neurological findings.

## Results

Adalimumab treatment can be effective for neurosarcoidosis.

## Conclusion

Management of neurosarcoidosis could be challenging despite usage of many immunosuppressant modalities.

## Disclosure of interest

None declared.

